# A genome-wide association study identifies *WT1* variant with better response to 5-fluorouracil, pirarubicin and cyclophosphamide neoadjuvant chemotherapy in breast cancer patients

**DOI:** 10.18632/oncotarget.5837

**Published:** 2015-11-09

**Authors:** Lina Wu, Lu Yao, Hong Zhang, Tao Ouyang, Jinfeng Li, Tianfeng Wang, Zhaoqing Fan, Tie Fan, Benyao Lin, C. Cameron Yin, Yuntao Xie

**Affiliations:** ^1^ Breast Center, Key Laboratory of Carcinogenesis and Translational Research, Ministry of Education, Beijing Cancer Hospital & Institute, Peking University Cancer Hospital, Beijing, P. R. China; ^2^ Central Laboratory, Key Laboratory of Carcinogenesis and Translational Research, Ministry of Education, Beijing Cancer Hospital & Institute, Peking University Cancer Hospital, Beijing, P. R. China; ^3^ Department of Hematopathology, The University of Texas MD Anderson Cancer Center, Houston, TX, USA

**Keywords:** WT1, rs1799937, breast cancer, anthracycline, neoadjuvant chemotherapy

## Abstract

Breast cancer is believed to result from the interplay of genetic and non-genetic risk factors, and individual genetic variation may influence the efficacy of chemotherapy. Here we conducted a genome-wide association study to identify single nucleotide polymorphisms (SNPs) associated with response to anthracycline- and taxane-based neoadjuvant chemotherapy in breast cancer patients. In the discovery stage, we divided 92 patients who received anthracycline-based neoadjuvant chemotherapy into 2 groups according to pathologic response and performed a genome-wide study using Affymetrix SNP6.0 genechip. Of 389,795 SNPs associated with pathologic complete response (pCR), we identified 2 SNPs, rs6044100 and rs1799937, that were significantly associated with pCR after neoadjuvant chemotherapy. In the validation stage, genotype analysis of samples from an independent cohort of 401 patients who received anthracycline-based neoadjuvant regimens and 467 patients who received taxane-based regimens was performed using sequencing analysis. We found that only SNP rs1799937, located in the *WT1* gene, was associated with pCR after anthracycline-based neoadjuvant therapy (AA vs GG; odds ratio [OR], 2.81; 95% confidence interval [CI], 1.13–6.98; *P* < 0.05) but not after taxane-based neoadjuvant therapy (AA vs GG; OR, 0.85; 95% CI, 0.36–2.04; *P* = 0.72). These results suggest that *WT1* may be a potential target of anthracycline-based neoadjuvant therapy for breast cancer.

## INTRODUCTION

Breast cancer is the most common malignant neoplasm among women in the world. It is believed to result from a complex interplay of genetic and non-genetic risk factors. Linkage and family-based studies have shown that germline mutations of high- and moderate-penetrance genes such as *BRCA1/2*, *PTEN*, *ATM*, *CHEK2*, *TP53*, *PALB2*, and *BRIP1* account for 20%–25% of the genetic component of breast cancer [1]. Genetic association studies, such as genome-wide association studies (GWASs), have identified low-penetrance common variants (predominantly single nucleotide polymorphisms [SNPs]) associated with breast cancer risk. Since the first 3 GWASs that identified SNPs associated with breast cancer susceptibility were conducted in early 2007 [2–4], over 80 loci associated with increased breast cancer risk have been found [5–16]. These studies have suggested that high- and moderate-penetrance genes and GWAS-identified low-penetrance common variants together may explain about 50% of the familial risk of breast cancer [17].

Accumulating evidence has shown that certain genetic variations are associated with response to chemotherapy in patients with lung cancer [18, 19]. However, genetic variants associated with tumor response to specific chemotherapy regimens in breast cancer have rarely been studied [20–22]. Neoadjuvant chemotherapy is increasingly used for operable primary breast cancer because it yields survival rate at least equal to those of adjuvant chemotherapy [23–25]. Some of the most effective cytotoxic agents against breast cancer are the anthracyclines, which have formed the backbone of most adjuvant and neoadjuvant regimens for more than 2 decades [23, 26–29]. Another class of effective cytotoxic agents, taxanes (such as paclitaxel and docetaxel), are also used as standard therapy for breast cancer, as monochemotherapy, in combination therapy, or in sequential therapy [30–32]. As neoadjuvant therapy, taxanes have produced dramatic increases in response rates [33–35]. Still, only a minority of patients achieve pathologic complete response (pCR) after receiving either anthracycline- or taxane-based neoadjuvant regimens. Given the lack of a preferred neoadjuvant treatment regimen, identifying which patients are most likely to respond to a specific regimen could significantly improve breast cancer treatment outcomes and facilitate the development of targeted therapies. Since GWASs have been successful in identifying low-penetrance common variants for breast cancer predisposition, several studies have investigated the potential roles of GWAS-identified SNPs in the choice of neoadjuvant therapy and evaluation of patient prognosis [18, 19]. In the current GWAS, we aimed to identify associations between SNPs associated with breast cancer susceptibility and response to anthracycline- and taxane-based neoadjuvant chemotherapy in patients with breast cancer.

## RESULTS

### Patient characteristics

The demographic and clinicopathologic characteristics of the 960 breast cancer patients who received neoadjuvant chemotherapy and were included in the discovery or validation cohort are shown in Table 1. In the discovery stage, the pCR rate was 32.6%. In the validation stage, the pCR rate was 15.5% in patients who received anthracycline-based neoadjuvant therapy and 17.1% in those who received a taxane-based regimen.

**Table 1:** Patient characteristics

Characteristic	No. of patients		Discoverycohort (*N* = 92)		Validation cohort				*P* value
					Anthracycline (*N* = 401)		Taxane (*N* = 467)		
	*N*	%	*N*	%	*N*	%	*N*	%	
Age (years)									
≤ 50	571	59.5	65	70.7	250	62.3	256	54.8	0.006*
> 50	389	40.5	27	29.3	151	37.7	211	45.2	
Tumor size									
≤ 2cm	381	40.0	22	23.9	147	37.3	212	45.5	< 0.001*
> 2cm	571	60.0	70	76.1	247	62.7	254	54.5	
Unknown	8		0		7		1		
Tumor grade									
I	95	10.3	6	6.7	45	11.6	44	9.8	0.21
II	691	74.7	68	76.4	277	71.2	346	77.4	
III	139	15.0	15	16.9	67	17.2	57	12.8	
Unknown	35		3		12		20		
Lymph node involvement									
Negative	525	55.4	63	69.2	230	57.6	232	50.7	0.002*
Positive	423	44.6	28	30.8	169	42.4	226	49.3	
Unknown	12		1		2		9		
Estrogen receptor status									
Negative	377	39.4	44	48.4	155	38.8	178	38.2	0.18
Positive	579	60.6	47	51.6	244	61.2	288	61.8	
Unknown	4		1		2		1		
Progesterone receptor status									
Negative	479	50.2	51	56.7	206	51.6	222	47.7	0.23
Positive	475	49.8	39	43.3	193	48.4	243	52.3	
Unknown	6		2		2		2		
HER2 status									
Negative	694	72.4	56	61.5	296	73.8	342	73.4	0.05
Positive	264	27.6	35	38.5	105	26.2	124	26.6	
Unknown	2		1		0		1		
Surgery type									
BCS	406	42.3	51	55.4	171	42.6	184	39.4	0.017*
Mastectomy	554	57.7	41	44.6	230	57.4	283	60.6	
Chemotherapy cycles									
≥ 4	897	93.4	92	100.0	361	90.0	444	95.1	< 0.001*
< 4	63	6.6	0	0.0	40	10.0	23	4.9	
Adjuvant chemotherapy									
No	332	34.6	15	16.3	113	28.2	204	43.7	< 0.001*
Yes	628	65.4	77	83.7	288	71.8	263	56.3	
Adjuvant endocrine therapy									
No	395	41.1	35	38.0	162	40.4	198	42.4	0.68
Yes	565	58.9	57	62.0	239	59.6	269	57.6	
Pathologic complete response									
non-pCR	788	82.1	62	67.4	339	84.5	387	82.9	< 0.001*
pCR	172	17.9	30	32.6	62	15.5	80	17.1	

HER2, human epidermal growth factor receptor 2; BCS, breast-conserving surgery; pCR, pathologic complete response

* A *P* value < 0.05 was considered statistically significant.

### Identification of SNPs

**Figure 1:**
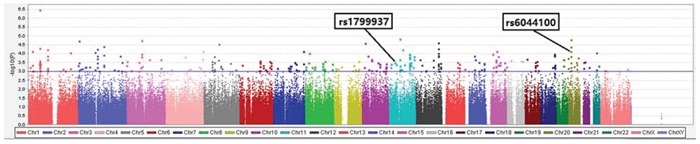
Scatter plot of *P* values (shown as -log10) in association test of 389,795 single nucleotide polymorphismalleles with pathologic response status at the end of anthracycline-based neoadjuvant chemotherapy in discovery cohort of 92 breast cancer patients. Colors indicate chromosomes

The discovery cohort included 92 patients who each received 4 cycles of the anthracycline-based CTF neoadjuvant regimen. pCR was used to differentiate cases from controls. All individuals had < 10% of missing genotypes, and all were genotyped as females based on X-chromosome genotypes, and therefore all were included. GWAS was performed using PLINK on genotype data from 30 patients with pCR and 62 patients with non-pCR. Of 389,795 SNPs included in the analysis, 351 SNPs showed significant associations with pCR. We then employed 3 additional criteria to select SNPs for inclusion in the validation stage. First, the 351 SNPs that showed significant association with pCR were confirmed using a genome-wide significance at *P* < 1e-03 (Figure 1). Second, the data set was narrowed down to 32 SNPs that are known to be related to genes that correlate with cancer using a search of gene annotation databases and published literature (ORs ranging from 2.89 [95% CI, 1.53–5.48] to 6.14 [95% CI, 2.07–18.20]) (Table 2). Third, using unadjusted univariate Cox proportional hazard models (*P* < 0.05), we ended up with only 2 SNPs with a pCR rate that was significantly higher for both homozygotes (Table 3).

**Table 2:** SNPs significantly associated with breast cancer in patients who received anthracycline-based CTF chemotherapy regimens in discovery cohort

						MAF			
SNP	Minor allele	Chr.	Position	Gene	Location	pCR	non-pCR	OR (95%CI)	*P* value
rs6135795	G	20	16534948	KIF16B	Intron	0.62	0.28	4.07 (2.11–7.83)	1.61 × 10^−5^*
rs4748316	T	10	16779881	RSU1	Intron	0.48	0.18	4.17 (2.10–8.27)	2.55 × 10^−5^*
rs6686072	C	1	57936193	DAB1	Intron	0.40	0.13	4.33 (2.07–9.06)	4.98 × 10^−5^*
rs687660	A	11	70111532	PPFIA1	Upstream	0.32	0.08	5.10 (2.19–11.87)	5.97 × 10^−5^*
rs3102072	A	1	20964066	PINK1	Intron	0.62	0.31	3.61 (1.89–6.90)	7.39 × 10^−5^*
**rs6044100**	**T**	**20**	**16524972**	**KIF16B**	**Intron**	**0.42**	**0.15**	**4.05 (1.98–8.29)**	7.65 × 10^−5^*
rs9806453	G	15	60943845	RORA	Intron	0.67	0.37	3.46 (1.80–6.64)	1.43 × 10^−4^*
rs6075070	G	20	16536424	KIF16B	Intron	0.42	0.16	3.80 (1.87–7.72)	1.44 × 10^−4^*
rs4811431	T	20	52031569	TSHZ2	Intron	0.28	0.08	4.88 (2.02–11.77)	1.78 × 10^−4^*
rs4580153	T	16	81817239	PLCG2	Intron	0.32	0.10	4.17 (1.86–9.35)	2.84 × 10^−4^*
rs12481468	C	20	43532438	YWHAB	intron	0.22	0.04	6.14(2.07–18.20)	3.16 × 10^−4^*
**rs1799937**	**A**	**11**	**32410774**	**WT1**	**Intron**	**0.47**	**0.21**	**3.33 (1.70–6.51)**	3.38 × 10^−4^*
rs1004895	A	20	16540600	KIF16B	Intron	0.40	0.16	3.54 (1.74–7.22)	3.38 × 10^−4^*
rs32496	A	5	55641639	MAP3K1	Upstream	0.68	0.40	3.24 (1.68–6.23)	3.38 × 10^−4^*
rs16944877	C	12	115459615	TBX3	Upstream	0.35	0.13	3.77 (1.77–8.04)	3.74 × 10^−4^*
rs12530912	G	7	116127529	CAV2	Intron	0.28	0.08	4.35 (1.85–10.25)	3.96 × 10^−4^*
rs17191246	C	15	60386336	ANXA2	Downstream	0.27	0.08	4.49 (1.85–10.90)	4.56 × 10^−4^*
rs10443217	G	1	57979666	DAB1	Intron	0.38	0.14	3.64 (1.73–7.66)	4.58 × 10^−4^*
rs1620195	T	12	32084747	H3F3C	Upstream	0.42	0.18	3.37 (1.68–6.76)	4.58 × 10^−4^*
rs10493224	A	1	57938752	DAB1	Intron	0.25	0.07	4.67 (1.85–11.77)	5.14 × 10^−4^*
rs4833326	C	4	126424441	FAT4	Downstream	0.67	0.40	3.00 (1.57–5.74)	7.41 × 10^−4^*
rs1467089	T	8	49960530	SNAI2	Upstream	0.40	0.17	3.27 (1.61–6.62)	7.52 × 10^−4^*
rs12151836	G	2	86601052	KDM3A	Upstream	0.28	0.09	3.92 (1.70–9.04)	8.24 × 10^−4^*
rs17588172	G	7	116154015	CAV2	Downstream	0.28	0.09	3.92 (1.70–9.04)	8.24 × 10^−4^*
rs4631527	G	9	110270036	KLF4	Upstream	0.57	0.31	2.93 (1.55–5.57)	8.29 × 10^−4^*
rs10857592	C	10	49732975	ARHGAP22	Intron	0.24	0.07	4.46 (1.75–11.36)	9.02 × 10^−4^*
rs1080954	T	20	16506016	KIF16B	Intron	0.47	0.23	3.01 (1.55–5.85)	9.07 × 10^−4^*
rs3821629	C	3	25620132	RARB	Intron	0.52	0.27	2.94 (1.54–5.62)	9.17 × 10^−4^*
rs3019286	A	8	99895405	STK3	Intron	0.60	0.34	2.89 (1.53–5.48)	9.59 × 10^−4^*
rs12039126	A	1	56681381	PPAP2B	Downstream	0.27	0.08	4.00 (1.69–9.49)	9.73 × 10^−4^*
rs7307249	G	12	117077849	MAP1LC3B2	Downstream	0.37	0.15	3.28 (1.59–6.78)	9.80 × 10^−4^*
rs527912	A	1	20934283	CDA	Intron	0.43	0.20	3.06 (1.55–6.03)	9.85 × 10^−4^*

SNP, single nucleotide polymorphism; CTF, 5-fluorouracil, pirarubicin, and cyclophosphamide; Chr., chromosome; MAF, minor allele frequency; pCR, pathologic complete response; OR, odds ratio; CI, confidence interval

* A *P* value < 0.05 was considered statistically significant.

The two SNPs examined in the validation phase were marked by bold.

**Table 3:** SNPs for which homozygosity was significantly associated with pCR in the discovery cohort

SNP	Genotype	No. (*N* = 92)	%	Pathologic response				*P* value
				non-pCR (*N* = 62)		pCR (*N* = 30)		
				No.	%	No.	%	
rs1799937	A A	10	10.9	4	40.0	6	60.0	0.002*^a^
	G A	34	37.0	18	52.9	16	47.1	
	G G	48	52.2	40	83.3	8	16.7	
rs6044100	C C	55	59.8	46	83.6	9	16.4	< 0.001*^b^
	T C	30	32.6	14	46.7	16	53.3	
	T T	7	7.6	2	28.6	5	71.4	

SNP, single nucleotide polymorphism; pCR, pathologic complete response

* A *P* value < 0.05 was considered statistically significant

a AA vs GG in rs11799937

b CC vs TT in rs6044100.

One of these two SNPs, rs1799937, is located in an intron of the *WT1* gene at 11p13; the A allele had an OR of 3.33 (95% CI, 1.70–6.51) compared with the G allele. The other SNP, rs6044100, is located in an intron of the *KIF16B* gene at 20p11, and the T allele had an OR of 4.05 (95% CI, 1.98–8.29) compared with the C allele. We found significantly higher pCR rates in patients with the rs1799937 AA genotype (60.0%) and the rs6044100 TT genotype (71.4%) than in patients with the rs1799937 GG genotype (16.7%) and the rs6044100 CC genotype (16.4%) (Table 3).

### Association of rs1799937genotype and response to anthracycline-based regimens

Having confirming that the SNPs rs1799937 AA genotype and rs6044100 TT genotype are associated with pCR, we investigated the potential association between these two genetic variants and pCR after anthracycline-based neoadjuvant chemotherapy. We attempted to validate rs1799937 and rs6044100 in an independent cohort of 401 patients who received CTF regimens by sequencing analysis. The results showed that patients with the rs1799937 AA genotype benefited from anthracycline-based neoadjuvant chemotherapy more than patients with the GG genotype; the pCR rates of the AA versus GG genotypes were 26.7% and 11.5%, respectively (*P* = 0.035). Logistic regression models showed an odds ratio for patients with the rs1799937 AA genotype compared with those with the GG genotype of 2.81 (95% CI, 1.13–6.98; *P* = 0.026, Table 4). However, no association was identified between rs6044100 and pCR; the pCR rates after CTF therapy for patients with the TT genotype versus those with the CC genotype were 18.8% and 15.2%, respectively (*P* = 0.86).

**Table 4:** Associations between SNP genotypes and pCR in 401 validation cohort patients who underwent anthracycline-based (CTF) chemotherapy. Two SNPs were examined

SNP	Genotype	No. (*N* = 401)	%	Pathologic response				*P* value	OR (95%CI)	*P* value
				non-pCR (*N* = 339)		pCR (*N* = 62)				
				No.	%	No.	%			
rs1799937	A A	30	7.5	22	73.3	8	26.7	0.035*	2.81(1.13–6.98)	0.026*^a^
	G A	153	38.2	124	81	29	19		1.81(1.01–3.23)	0.046*^b^
	G G	218	54.4	193	88.5	25	11.5			
rs6044100	C C	224	57.3	190	84.8	34	15.2	0.86	0.78(0.30–2.03)	0.60^c^
	T C	135	34.5	113	83.7	22	16.3		0.84(0.31–2.29)	0.74^d^
	T T	32	8.2	26	81.2	6	18.8			
	Unknown	10		10		0				

SNP, single nucleotide polymorphism; pCR, pathologic complete response; CTF, 5-fluorouracil, pirarubicin, and cyclophosphamide; OR, odds ratio; CI, confidence interval

* A *P* value of < 0.05 was considered statistically significant.

a AA vs GG

b AG vs GG

c CC vs TT

d CT vs TT.

### Association between rs1799937genotype and response to taxane-based regimens

Further, we explored the association of rs1799937 with pCR in patients receiving taxane-based neoadjuvant chemotherapy. Rs1799937 genotypes were assessed by sequencing in an independent cohort of 467 patients who received taxane-based regimens. No association was observed between rs1799937 genotype and pCR status; the two homozygous genotypes showed similar pCR rates (17.1% vs 19.5%; *P* = 0.34, Table 5).

**Table 5:** Associations between SNP genotypes and pCR in 467 validation cohort patients who underwent taxane-based chemotherapy

SNP	Genotype	No.(*N* = 467)	%	Pathologic response				*P* value	OR (95%CI)	*P* value
				non-pCR		pCR				
				No. (*N* = 387)	%	No.(*N* = 80)	%			
rs1799937	A A	41	8.8	34	82.9	7	17.1	0.34	0.85 (0.36–2.04)	0.72^a^
	G A	185	39.6	159	85.9	26	14.1		0.68 (0.40–1.14)	0.14^b^
	G G	241	51.6	194	80.5	47	19.5			

pCR, pathologic complete response; SNP, single nucleotide polymorphism; OR, odds ratio; CI, confidence interval

* A *P* value of < 0.05 was considered statistically significant.

a AA vs GG

b GA vs GG.

### Association between rs1799937genotype and survival

**Figure 2:**
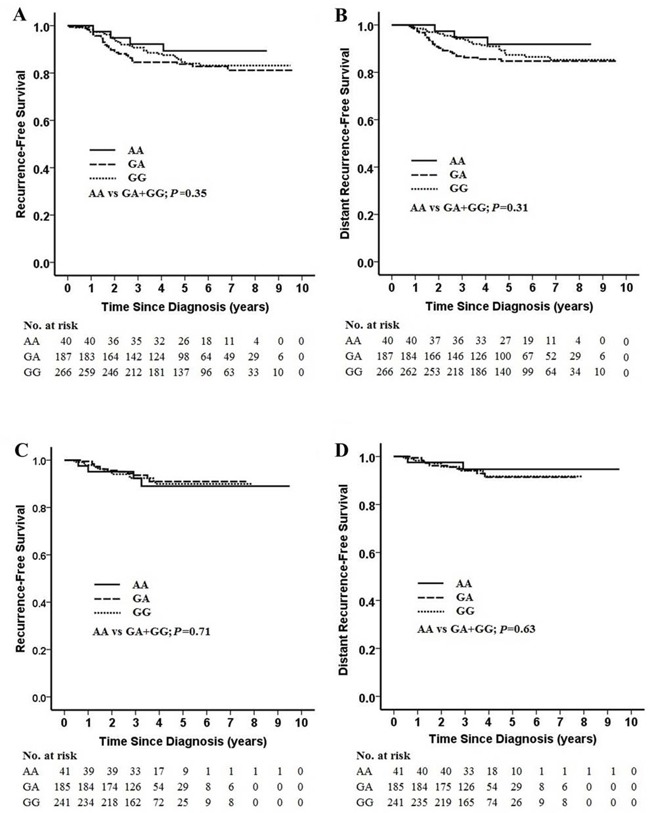
Kaplan-Meier estimates of recurrence-free survival (RFS) and distant recurrence-free survival (DRFS) by rs1799937 genotype and neoadjuvant chemotherapy regimen **Panel A.** 10-year RFS by rs1799937 genotype in patients receiving anthracycline-based regimen. **Panel B.** 10-year DRFS by rs1799937 genotype in patients receiving anthracycline-based regimen. **Panel C.** 10-year RFS by rs1799937 genotype in patients receiving taxane-based regimen. **Panel D.** 10-year DRFS by rs1799937 genotype in patients receiving taxane-based regimen.

Among the 493 patients who received CTF regimen, including 92 from the discovery stage and 401 from the validation stage, patients with the rs1799937 AA genotype had a slightly higher but not statistically significant 5-year recurrence-free survival (RFS) rate (89.3%; 95% CI, 79.5%-99.1%; AA vs GA+GG; *P* = 0.35) and 5-year distant recurrence-free survival (DRFS) rate (92.2%; 95% CI, 83.8%-100.1%; AA vs GA+GG; *P* = 0.31) than those with the rs1799937 GG genotype (RFS, 84.0%; 95% CI, 79.1%-88.9%; DRFS, 87.4%; 95% CI, 82.9%-91.9%; Figures 2a and 2b). However, no difference in RFS and DRFS was detected between rs1799937 AA versus GG genotype in the 467 breast cancer patients treated with taxane-based neoadjuvant chemotherapy (Figures 2c and 2d).

## DISCUSSION

We performed a GWAS to assess genetic variants that may be associated with pCR in patients receiving neoadjuvant chemotherapy for breast cancer. We identified 1 SNP, rs1799937 A > G, as a genetic variant associated with response to anthracycline-based CTF neoadjuvant chemotherapy. Patients with the rs1799937 AA genotype showed significantly higher rates of pCR and better prognosis after treatment with CTF than patients with the GG genotype. However, we found that the AA allele of rs1799937 was not associated with sensitivity to taxane-based neoadjuvant chemotherapy regimens.

The rs1799937 A > G SNP is located in an intron region of the *WT1* gene at 11p13. *WT1* encodes a transcription factor that contains 4 zinc-finger motifs at the C-terminus and plays an important role in organ development and cell survival [36]. In addition to regulating nuclear transcription, *WT1* is also involved in RNA splicing and metabolism [37, 38]. *WT1* was first identified as a tumor suppressor gene in Wilms' tumor. However, accumulating evidence has shown that overexpression of *WT1* exerts an oncogenic effect in other tumors [36, 39, 40] and is linked to poor prognoses in leukemia and breast cancer [41, 42].

Previous studies have also shown that *WT1* gene expresion is related to sensitivity to chemotherapy. Perugorria et al. have reported that *WT1* knockdown markedly sensitized hepatocellular carcinoma cell lines to doxorubicin-induced apoptosis [43]. Interestingly, the effect of *WT1* expression on apoptosis of hepatocellular carcinoma cells seems to be specific to doxorubicin, and not to other chemotherapeutic agents. Doxorubicin, an anthracycline, is a potent inhibitor of Top2 isozymes and exerts its genotoxic effect by impeding DNA duplication [44–36]. We speculate that rs1799937 may function by altering *WT1* gene expression, thus rendering cancer cells in patients with the AA allele more sensitive to anthracycline-based chemotherapy.

In summary, our results indicate that anthracycline-based neoadjuvant chemotherapy may benefit breast cancer patients with the rs1799937 AA allele more than those with the GG allele. Therefore, further investigation of *WT1* as a potential target of chemotherapy for breast cancer is warranted.

## MATERIALS AND METHODS

### Patient selection

The GWAS included 960 patients with breast cancer who were treated at Peking University Cancer Hospital between October 2003 and November 2011. Written informed consent was obtained from all patients, and this study was approved by the Research and Ethical Committee of Peking University Cancer Hospital.

The discovery stage included 92 patients from a pool of 493 patients who had received 4 cycles of an anthracycline-based neoadjuvant regimen consisting of cyclophosphamide, pirarubicin (THPADM), 5-fluorouracil (CTF). This regimen included pirarubicin 35 mg/m^2^ i.v. on day 1 and day 8, cyclophosphamide 500 mg/m^2^ i.v. on day 1 and day 8, and 5-fluorouracil 200 mg/m^2^/day i.v. continuous infusion for 28 days, every 4 weeks. All the 92 patients received an identical CTF regimen with the same dosage and same number of cycles. There was no bias in terms of any patient characteristics. Using pCR, defined as the absence of invasive breast tumor cells in the breast and axillary lymph nodes after completion of neoadjuvant chemotherapy, the patients were divided into 2 pathologic response groups with 30 in the pCR group and 62 in the non-pCR group. In the validation stage, 401 patients who had received 2–6 cycles of CTF and 467 patients who had received a taxane-based regimen using paclitaxel were included.

### Genotyping methods and quality control

In the discovery stage, we isolated DNA from peripheral blood lymphocytes using phenol-chloroform extraction and ultrapurification. Genotyping was conducted using Affymetrix Genome-Wide Human SNP Array 6.0 chips (Santa Clara, CA). We performed systematic quality control on the raw genotyping data to filter out unqualified samples and SNPs. As quality control, we excluded DNA samples that failed to be genotyped as female based on X-chromosome genotypes. SNPs were excluded if (1) they were not mapped on autosomal chromosomes; (2) they had a call rate < 95%; (3) they had minor allele frequency < 0.01; (4) they deviated from the Hardy-Weinberg equilibrium at *P* < 1e^−^05; or (5) their genotyping clusters had poor resolution. These led to a total of 389,795 SNPs to be analyzed using a GWAS toolset PLINK [47].

The genotypes of the patients with and without pCR were compared. We selected SNPs that were (1) associated with pCR in the discovery stage at *P* < 1e-03; (2) located in genes that have been correlated with cancer in gene annotation databases and published literature; and (3) associated with pCR status in the discovery stage at *P* < 0.05 using unadjusted univariate Cox proportional hazard models. Finally, 2 SNPs, rs1799937 and rs6044100, were identified.

For the validation phase, genomic DNA samples were extracted from peripheral blood lymphocytes of an additional 868 patients, including 401 who received anthracyclin-based regimens and 467 who received taxane-based regimens. Genotyping of the samples was conducted using a polymerase chain reaction/sequencing assay (ABI 3730 system, Applied Biosystems, Foster City, CA).

### Statistical analysis

Association between SNPs and response to neoadjuvant chemotherapy in patients with breast cancer was assessed using logistic regression models and reported as odds ratios (ORs) and 95% confidence intervals (CIs). ORs were estimated for heterozygotes and homozygotes for the variant allele compared with homozygotes for the common allele. A logistic regression model was applied to determine whether a factor was an independent predictor of pCR in a multivariate analysis. Survival analysis was performed using the Kaplan-Meier curves. All statistical tests were two-sided. All statistical analyses were performed using SPSS 16.0 software (SPSS Inc., Chicago, IL).

## SUPPLEMENTARY TABLES AND FIGURE



## Abbreviations

GWAS: genome-wide association study
SNP: single nucleotide polymorphism
pCR: pathologic complete response
CTF: 5-fluorouracil, pirarubicin, and cyclophosphamide
OR: odds ratio
CI: confidence interval
RFS: recurrence-free survival
DRFS: distant recurrence-free survival
