# Supplementary material for: A genome-wide association study identifies WT1 variant with better response to 5-fluorouracil, pirarubicin and cyclophosphamide neoadjuvant chemotherapy in breast cancer patients
Source: Oncotarget. 2015 Nov 9;7(4):5042–52. doi: 10.18632/oncotarget.5837 (PMC4826264; doi:10.18632/oncotarget.5837)
Supplement: Supplementary file 1 [file oncotarget-07-5042-s001.pdf]

## SUPPLEMENTARY TABLES AND FIGURE

**Supplementary Table S1: Associations between SNP genotypes and pCR in 493 patients who underwent anthracycline-based (CTF) chemotherapy**

| SNP | Genotype | No. (*N* = 493) | % | Pathologic response non-pCR (*N* = 401) No. | % | pCR (*N* = 92) No. | % | *P* value | OR (95%CI) | *P* value |
|---|---|---|---|---|---|---|---|---|---|---|
| rs1799937 | A A | 40 | 8.1 | 26 | 65 | 14 | 35 | <0.001* | 3.80(1.80-8.01) | <0.001[*a] |
| | G A | 187 | 37.9 | 142 | 75.9 | 45 | 24.1 | | 2.24(1.36-3.67) | <0.001[*b] |
| | G G | 266 | 54 | 233 | 87.6 | 33 | 12.4 | | | |
| rs6044100 | C C | 279 | 57.8 | 236 | 84.6 | 43 | 15.4 | 0.045* | 0.46(0.21–1.00) | 0.05[c] |
| | T C | 165 | 34.2 | 127 | 77 | 38 | 23 | | 0.76(0.35–1.67) | 0.50[d] |
| | T T | 39 | 8.1 | 28 | 71.8 | 11 | 28.2 | | | |
| | Unknown | 10 | | | | | | | | |

SNP, single nucleotide polymorphism; pCR, pathologic complete response
A *P* value < 0.05 was considered statistically significant. [a] AA vs GG in rs11799937; [b] GA vs GG in rs11799937; [c]CC vs TT in rs6044100; [d]TC vs TT in rs6044100.

**Supplementary Table S2: Relationship between *WT1* rs1799937 genotype and *WT1* gene expression**

| SNP | Genotype | No. (*N* = 127) | % | Expression | | | | *P* value |
|---|---|---|---|---|---|---|---|---|
| | | | | Low (*N* = 83) | | High (*N* = 44) | | |
| | | | | No. | % | No. | % | |
| rs1799937 | A A | 12 | 9.7 | 8 | 66.7 | 4 | 33.3 | 0.97 |
| | G A | 43 | 34.7 | 29 | 67.4 | 14 | 32.6 | |
| | G G | 69 | 55.6 | 45 | 65.2 | 24 | 34.8 | |
| | Unknown | 3 | | 1 | | 2 | | |

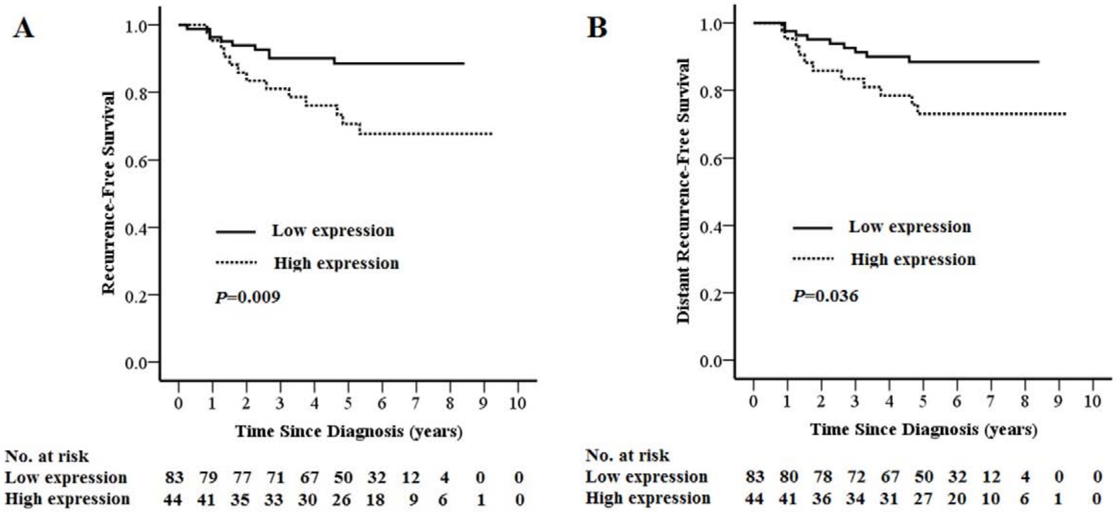


**Supplementary Figure S1: Kaplan-Meier analysis of recurrence-free survival (RFS) and distant recurrence-free survival (DRFS) by WT1 expression in 127 patients receiving CTF regimen.** **Panel A.** 10-year RFS by WT1 expression. **Panel B.** 10-year DRFS by WT1 expression.
